# Efficacy and antitumor activity of a mutant type of interleukin 2

**DOI:** 10.1038/s41598-022-09278-7

**Published:** 2022-03-30

**Authors:** Rada Dehghan, Arezoo Beig Parikhani, Sirous Zeinali, Mohamadali Shokrgozar, Amir Amanzadeh, Soheila Ajdary, Reza Ahangari Cohan, Yeganeh Talebkhan, Mahdi Behdani

**Affiliations:** 1 Venom and Biotherapeutics Molecules Laboratory, Department of Medical Biotechnology, Biotechnology Research Center, Pasteur Institute of Iran, 1316543551 Tehran, Iran; 2 Molecular Medicine Department, Biotechnology Research Center, Pasteur Institute of Iran, Tehran, Iran; 3 National Cell Bank of Iran, Pasteur Institute of Iran, Tehran, Iran; 4 Department of Immunology, Pasteur Institute of Iran, Tehran, Iran; 5 Department of Nanobiotechnology, New Technologies Research Group, Pasteur Institute of Iran, Tehran, Iran; 6 Department of Medical Biotechnology, Biotechnology Research Center, Pasteur Institute of Iran, 1316543551 Tehran, Iran

**Keywords:** Biotechnology, Cancer, Immunology

## Abstract

Interleukin-2 (IL-2) is an important cytokine in survival, expansion, function of CD8+ T cells and natural killer cells in immunotherapy of melanoma and renal cell carcinomas. Its severe toxicity following binding to its high affinity IL-2 receptor alpha (IL-2Rα) has restricted its application in cancer patients. In the present study, we investigated the antitumor efficacy and cytotoxicity of a mutated human IL-2 previously designed by selective amino acid substitutions, and its reduced affinity towards high-affinity IL-2Rα (CD25) was approved compared to the wild type IL-2 (wtIL-2). Furthermore, their ability to induce PBMC cell proliferation, and interferon-gamma secretion was compared. The mutant IL-2 also represented higher antitumor activity and more efficient cytotoxicity than wild type hIL-2. The developed mutant IL-2 can be an alternative tool in IL-2 associated immunotherapy of various cancers.

## Introduction

Interleukin-2 (IL-2), a member of the γ-chain cytokines with molecular weight of 15 kDa compromising 133 amino acids and four alpha-helices, is primarily produced by activated CD4+ and CD8+ T cells^1–6^. It has been considered an important cytokine in survival, growth, proliferation, and differentiation of T cells and natural killer (NK) cells^4,7^. It plays crucial roles in induction of cellular and humoral immunity through proliferation and activation of antigen-activated T cells, stimulation of NK cells, and homeostasis^5,8–10^. Aldesleukin (Proleukin^®^), a recombinant IL-2 analogue, is the first FDA-approved cytokine for immunotherapy of metastatic renal cell carcinoma and malignant melanoma^1,4,7,8^. However, intravenous administration of IL-2 has been associated with toxicity^11,12^.

Stimulatory and regulatory functions of IL-2 are mediated by binding to multi-subunit IL-2 receptor (IL-2R) complex consisting of IL-2Rα (CD25), IL-2Rβ (CD122), and IL-2Rγ (CD132) subunits. IL-2 receptors consist of three forms; low-affinity (IL-2Rα alone), intermediate-affinity (IL-2Rβγ), and high-affinity (IL-2Rαβγ)^1,6,13,14^. The alpha subunit of the receptor, the adaptor molecule in high affinity receptors, plays an important role in affinity towards IL-2 while β and γ subunits participate in signal transduction. Activated effector CD4+ T cells, CD8+ T cells, and regulatory T cells (Treg) constitutively express high-affinity receptor subunits^1,4^. In contrast, intermediate affinity receptors lacking CD25 subunit are usually expressed in naïve CD8, CD4/CD8 memory T cells, and NK cells^4,6,10,13–15^.

Low efficacy of IL-2 therapy in cancer patients is due to the short serum half-life, which requires administration of high doses of IL-2 (600,000 IU/kg; 0.037 mg/kg, every 8 h for a maximum of 14 doses) and consequently, severe toxicity and vascular leak syndrome can occur^1,4,16,17^. In addition, the interaction of IL-2 with its alpha receptor and expansion of regulatory T cells will activate immunosuppressive responses and reduce T cell-mediated anti-tumor activities^1,6,9,18^.

To overcome this problem, several variants of IL-2 were designed in which binding to IL2-Rα was reduced without affecting the affinity of IL-2 towards IL-2Rβ or γ compared to the native IL-2^1,4,19^. It has been well documented that mutations in two amino acids, R38 and F42, play important roles in reducing the affinity of IL-2 to CD25^3,19^. In vitro treatment of human peripheral blood mononuclear cells (PBMCs) with IL-2 variants has reduced the production of inflammatory cytokines, including IL-1β, TNFα/β, and IFN-γ^1,19^. This approach has shown promising results in reducing the IL-2 toxicity compared to the wild type IL-2 and can be effectively applied in immunotherapies^3,19^.

In the present study, the efficacy and cytotoxicity of a mutant form of IL-2 with reduced binding to the alpha subunit which was previously designed by selective amino acid substitutions, was evaluated compared to the wild type human IL-2 (hIL-2).

## Results

### Protein expression and purification

**Figure 1:**
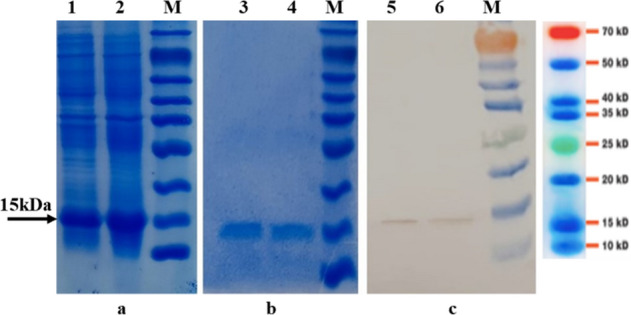
Analysis of protein expression: IPTG Induced bacterial lysate (**a**), Purified Protein (**b**) and western blotting analysis (**c**). #1, 3, 5: wtIL-2; #2, 4, 6: Mutant IL-2; M: Protein molecular weight marker. The mutant IL-2 protein was made by three amino acid substitutions (K35A, E61A, and F42A). The full-size original gels and the blot are presented in Supplementary Fig. S1a, S1b and S1c, respectively.

After expression and purification of recombinant wIL-2 and mutant IL-2, the proteins were analyzed on 12% SDS-PAGE (Fig. 1a,b and Supplementary Fig. 1a and 1b), and respective15kDa proteins were observed. The size of the proteins was confirmed by Western blotting analysis using an HRP-conjugated anti-His antibody (Fig. 1c and Supplementary Fig. 1c).

### Proliferation effect of recombinant IL-2 proteins on PBMCs

**Figure 2:**
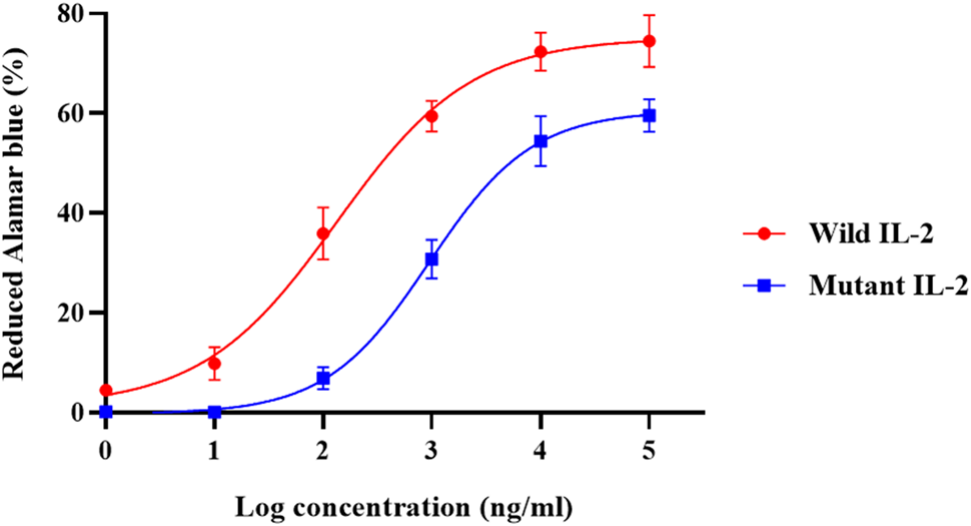
PBMC colorimetric proliferation assay. All experiments were done in triplicate. Data are represented as mean ± SD.

Cell proliferation assay was performed to determine the efficiency of wild and mutant IL-2 proteins on proliferation of PBMCs using Alamarblue. Both recombinant IL-2 proteins induced PBMC proliferation. However, a comparison of calculated EC50 values (133.3 and 944.8 ng/ml for wild and mutant IL-2, respectively) indicated that wIL-2 resulted in a significantly higher proliferation than the mutant protein (Fig. 2) (*P* < 0.0001).

### Effect of recombinant IL-2 proteins on IFN-γ production

**Figure 3:**
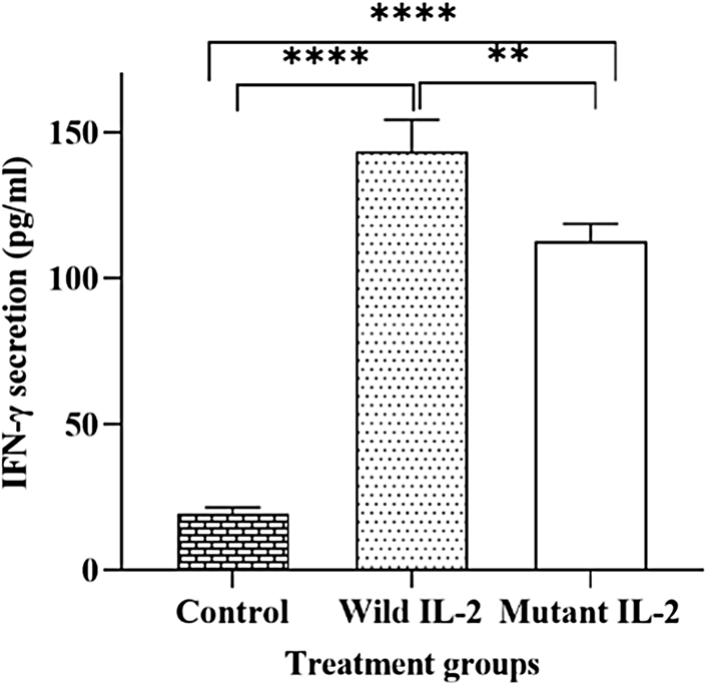
Assessment of IFN-γ secretion in IL-2 treated PBMCs. The results are mean ± SD values of triplicate experiments. Data comparisons were analyzed using ANOVA test (**P < 0.05; ****P < 0.0001).

To compare the effect of wild and mutant IL-2 proteins on IFN-γ secretion by T cells, PBMCs were stimulated with recombinant IL-2 proteins, and subsequently, IFN-γ level was measured by ELISA. The results revealed that IFN-γ secretion was significantly increased in response to both IL-2 proteins compared to the untreated control cells (*P* < 0.0001). Moreover, wIL-2 induced significantly higher levels of IFN-γ compared to mutant IL-2 (*P* < 0.05) (Fig. 3).

### Cytotoxicity assay

**Figure 4:**
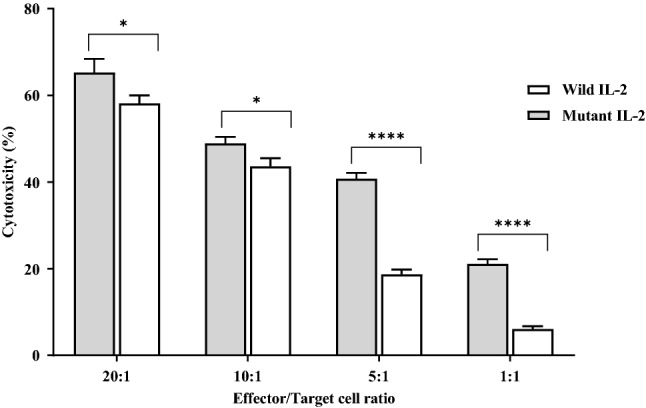
IL-2 cellular cytotoxicity effect in different effector and target cell ratios. All experiments were done in triplicate. Data analysis was done with t-test and represented as mean ± SD (*P < 0.03; ****P < 0.0001).

In a Calcein-AM mediated cytotoxicity assay, IL-2 stimulated effector PBMCs were co-cultured with K562 cell line. Cytotoxicity analysis revealed that increasing the effector to target cell ratio increased the cytotoxicity effect in comparison with the control group, particularly in ratios of 1:1 and 1:5, in which the observed cytotoxicity was significantly higher for cell complexes received mutant IL-2 protein rather than wild IL-2 (*P* < 0.0001). This finding demonstrated mutant IL-2 as a more effective molecule in inducing the cytotoxicity effect of PBMCs against K562 cell line (Fig. 4).

### In vivo antitumor activity of recombinant IL-2 proteins

**Figure 5:**
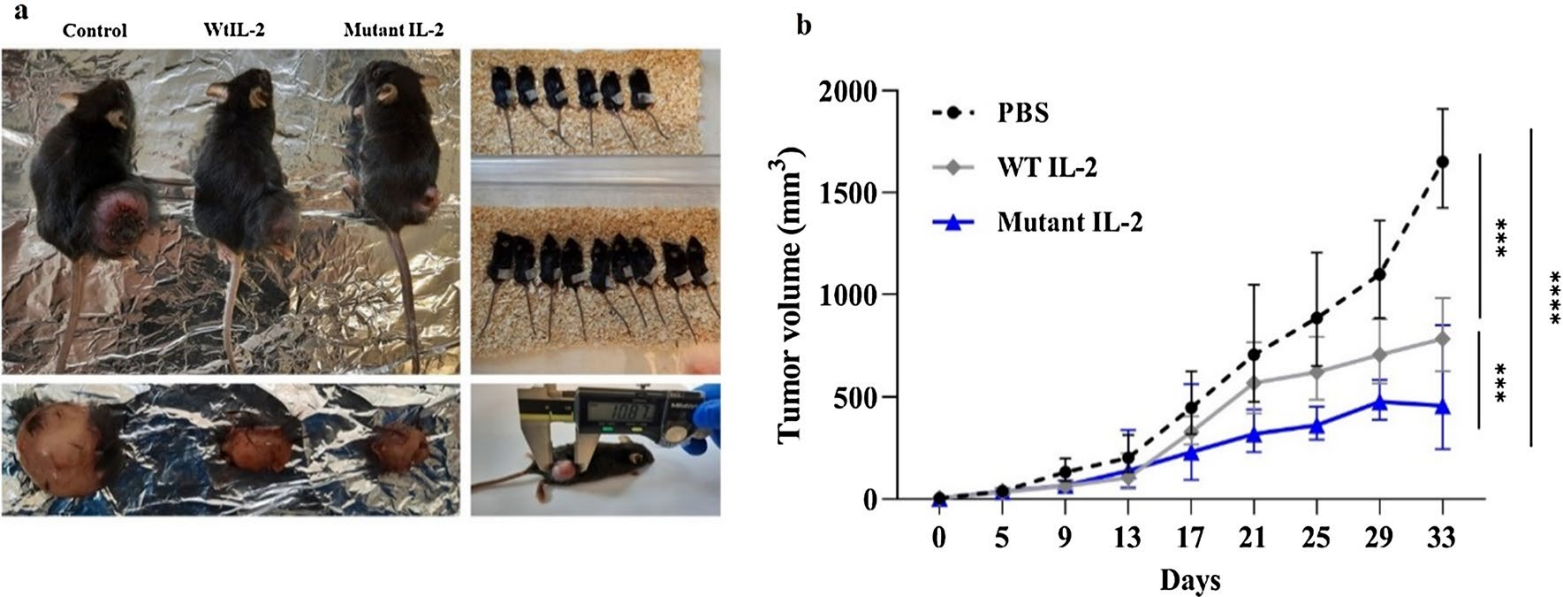
Assessment of in vivo antitumor activity: TC-1 cells (mouse lymphoblast B lymphocytes) were subcutaneously (s.c) injected into the right flank of the mice. 1 mg/kg of recombinant purified IL-2 proteins were injected at the tumor site two times per week for one month. (**a**) Macroscopic tumor sizes in individual mice from three tested groups; (**b**) Inhibited tumor volume in wild or mutant IL-2-treated mice vs. PBS treated control group. Data are represented as mean ± SD of tumor volume in treated groups (5 mice/group). (***P < 0.001, ****P < 0.0001).

To evaluate the antitumor activity of the expressed IL-2 proteins, tumor-bearing C57BL/6 mice were treated with 1 mg/kg of wild and mutant forms of IL-2 protein. The tumor size was measured for approximately one month after tumor transplantation, and tumor volume was calculated. It was observed that tumor growth in animals treated with wild or mutant IL-2 proteins was significantly inhibited compared to the PBS treated control mice (Fig. 5). Observations revealed that tumor volume of the mutant IL-2-treated mice was significantly decreased in comparison with the wild IL-2 treated group (*P* < 0.001).

## Discussion

Interleukin 2 plays a crucial role in immunotherapy of metastatic melanoma and renal cell carcinoma^4^. However, short half-life, severe toxicity, and activation of Tregs, which occur through binding to the high-affinity receptor limits its administration in clinic^1,19^. These side effects may be enhanced by the secretion of several inflammatory cytokines such as IL-1β, TNF-α, TNF-β, and IFN-γ in IL-2 stimulated PBMCs, which cause hypotension by producing nitric oxide as a mediator of vasodilatation^17,19^. In addition, these cytokines are involved in binding of IL-2 activated leukocytes to the endothelium by increasing the expression of adhesion molecules, yielding to pathogenic conditions such as vascular leak syndrome^19,20^.

Several approaches have been used to reduce the cytotoxicity of IL-2, including alterations in the route of administration and its combination with other medicines. Administration of anti-TNF antibodies in a murine model^3,21^ or the use of nitric oxide synthase inhibitors such as N-methyl arginine in dogs received intravenous IL-2 injection could decrease IL-2 side effects and hypotension^22^. Application of interleukin-2 variants is another approach that has attracted very attention during the last decades^1,3,4,6^.

In our previous study, potential amino acid substitutions which would reduce the affinity of IL-2 protein towards IL-2Rα subunit were identified based on computational studies^23^. Molecular dynamic simulations revealed that mutant 2 variant of IL-2 possessing triple mutations of K35A, E61A, and F42A had a significant reduced affinity to IL-2Rα which has been confirmed through flow cytometry analysis using anti-CD25 monoclonal antibody. This effect is in agreement with previous studies indicating preferentially stimulation of cytotoxic CD8+ T and NK cells, reduced interaction with Tregs and endothelial cells, lower toxicity, and improved immunity in comparison to wIL-2^1,3,6,19,24–26^. In the present study, the biological activity of a newly designed mutant form of human IL-2 with reduced affinity to CD25 was investigated and compared to the wild IL-2.

First, the effect of the mutant IL-2 on the proliferation of PBMCs and their IFN-γ secretion was examined. The results showed a reduced proliferation rate and IFN-γ secretion level compared to the wild IL-2 molecule. The weaker interaction of mutant IL-2 towards α-subunit could yield a lower proliferation effect on PBMCs, consistent with previous studies^19,27^.

Our finding is in line with that of Heaton et al. which reported low production of IFN-γ by PBMCs stimulated with a mutant IL-2 (R38A and F42K). They reported that this mutant molecule binds to the intermediate-affinity IL-2R and could induce cytotoxic effects on PBMCs^19^. Since IL-1β, TNF-α, and IFN-γ cytokines are believed to mediate cytotoxicity of IL-2 based immunotherapies^4,19^, mutant IL-2 with reduced binding to the high-affinity IL-2R may be more effective and less toxic for cancer treatment^19,28–30^.

IL-2 is a vital cytokine for effective maintenance and control of T cell responses and expansion of the CD8+ T cell population in in vivo conditions^24,31^. However, IL-2 administration to cancer patients has increased Treg cells at the tumor site which play a fundamental role in immune system inhibition and anti-tumor responses^6,24^. Our results indicated that despite a lower proliferation rate and IFN-γ production, the mutant IL-2 could induce a more cytotoxic effect in PBMCs against K562 cells than the wIL-2. To examine the toxicity of the molecule in in vivo conditions, mice were transplanted with TC-1 cells to induce tumoral tissue. The results revealed that local administration of recombinant IL-2 proteins could control the tumor growth, which was more prominent with mutant IL-2 than the wIL-2.

Levin et al., designed an IL-2 superkine with a decreased affinity toward IL-2Rα subunit^25^. They showed that superkine could induce superior expansion of cytotoxic T cells, improve anti-tumor responses in in vivo conditions, and elicit proportionally lesser expansion of Tregs and pulmonary oedema. In a similar study, Chen et al., reported that FSD13 mutant form of IL-2 was effective in NK92 cell line proliferation and activation of CD8+ T cells without considerable side effects or damages in lungs or liver of tested animals^6^. Our results are consistent with previous studies which used mutagenesis to reduce the binding of IL-2 molecule to the high-affinity IL-2R to improve its therapeutic effects^1,4,6,25,32^.

In conclusion, this study indicated that the mutant IL-2 (K35A, E61A, and F42A) could be a promising agent for IL-2-based immunotherapy of cancer. Further physiological studies regarding lung oedema as well as kidney and liver function are required to be evaluated.

## Materials and methods

### Ethics statement

All experiments and procedures were approved by the ethics committee of Pasteur Institute of Iran (IR.PII.REC.1400.034) and performed in accordance with the approved guidelines and regulations. Animal studies were performed in accordance with the ARRIVE reporting guidelines^33^. Human blood samples were obtained from one of the authors as a volunteer according to the same proposal approved by the ethics committee of Pasteur Institute of Iran and in accordance with the Declaration of Helsinki and other relevant guidelines, including the ethical guidelines for medical and health research on human subjects by the Iranian government. The informed consent was obtained before human blood sampling.

### Cloning, expression and purification of wild and mutant hIL-2

The amino acid sequence of the wild IL-2 was obtained from drug bank (Accession No. DB00041). In our previous study, IL-2 mutant molecules were designed through computational approaches. In brief, the amino acid residues involved in electrostatic interactions between IL-2 and IL-2Rα subunit were identified and alanine mutations were applied at selected positions and the variant with the lowest affinity towards IL-2Rα (M2 molecule, K35A, E61A, and F42A) was identified using docking, molecular dynamics simulation, umbrella sampling, and Gibbs energy calculations^23^. The two IL-2 encoding gene fragments were separately subcloned into pET28a expression vector at *NcoI* and *HindIII* restriction sites. The identity of the gene fragments was confirmed through restriction digestion and sequencing.

*E. coli* BL21 (DE3) strain was transformed with recombinant vectors and inoculated into 200 ml Luria Bertani (LB) medium. The expression of wild and mutant hIL-2 proteins was induced with 0.5 mM isopropyl β-D-1-thiogalactoside (IPTG) at OD600nm of 0.5 and incubated for additional 6 h. The bacterial pellet was resuspended in lysis buffer I (50 mM NaH_2_PO_4_, 300 mM NaCl, 10 mM Imidazole; pH8.0). The cells were sonicated at 100% amplitude (30 s pulses with 10 s intervals) and centrifuged at 10,000×*g* for 20 min. The pellet, containing inclusion bodies, was solubilized in lysis buffer II (50 mM NaH_2_PO_4_, 300 mM NaCl, 10 mM Imidazole, 8 M Urea; pH8.0) and placed under agitation for 1 h. After centrifugation (10,000×*g* for 30 min), the supernatant was loaded to Ni–NTA agarose column (ABT, Spain) at a flow rate of 1 ml/min. Refolding of the proteins was carried out through a gradient of urea concentration (from 8 M to 0) using the refolding buffer (50 mM NaH_2_PO_4_, 500 mM NaCl, 20 mM Imidazole; pH8.0). Histidine-tagged protein was eluted by the elution buffer (50 mM NaH_2_PO_4_, 500 mM NaCl, 250 mM imidazole; pH8.0). Protein concentration was determined by UV adsorption at 280 nm using a spectrophotometer (BioTeK, USA). The purity of the eluted protein was analyzed on 12% SDS-PAGE and Coomassie Brilliant Blue staining.

For western blotting, proteins were transferred to nitrocellulose membrane. After blocking the membrane with 3% w/v skim milk in phosphate-buffered saline (PBS) overnight (o/n) at 4 °C, the membrane was washed with PBS supplemented with 1% Tween-20 for 5 min (3 times). The membrane was treated with 1:2000 dilution of HRP-conjugated anti-His antibody, and the protein bands were visualized using 3,3 diaminobenzidine tetrahydrochloride (DAB) solution as the substrate.

### PBMC proliferation assay

Alamarblue assay was used to evaluate the proliferative responses of lymphocytes to the recombinant proteins. Heparinized peripheral blood was obtained from a healthy volunteer after approval by the Ethics committee of Pasteur Institute of Iran (IR.PII.REC.1400.034) according to the declaration of Helsinki and other relevant guidelines. PBMCs were isolated on a Ficoll-Hypaque (Lymphoprep, Nyegaard, Norway) density gradient approach^17^. PBMCs were washed and resuspended in RPMI-1640 medium supplemented with 100 U/ml penicillin, 100 mg/ml streptomycin (Invitrogen, UK), 2 mM L-glutamine, and 10% fetal calf serum (FCS, Sigma). The cells were seeded at a density of 2 × 10^5^ cells/well and stimulated with 2 µg/well of concanavalin A (Con A, Sigma). Negative control cells were treated with medium without any mitogen. The cells were incubated at 37 °C, 5% CO_2_ for 24 h. Subsequently, wIL-2 or mutant IL-2 proteins were added to the cells in a range of 0.01 nM to 10 µM and incubation continued for 48 h. Then, Alamarblue reagent was added to the wells and incubated for 16 h. Replicates of each experiment were analyzed on a microplate reader (BioTek ELx808, USA) at 570 and 600 nm. The percentage of reduction in Alamar blue was calculated according to the Manufacturer’s recommendations (Thermo Fisher Scientific, USA).

### IFN-γ assay

Isolated PBMCs were seeded at a density of 2 × 10^6^ cells/ml in 24-well plates, treated with 1 µg IL-2 proteins and incubated at 37 °C for 48 h. The supernatants were harvested and IFN-γ secretion level was measured using the Human IFN-γ ELISA kit (Karmania Pars Gene, IRAN).

### Cytotoxicity assay

Cytotoxicity assay was performed using Calcein-AM bio-detergent. Isolated PBMCs from Healthy donor were served as effector cells (E) against the target cell line (T), K562 (Human leukemia cell). In brief, PBMCs were stimulated with 1 µg mutant IL-2 or wtIL-2 for 48 h. Then, 1 × 10^6^ K562 cells/ml were labeled with 2 µM Calcein-AM (R&D Systems, USA) in complete RPMI-1640 medium, washed twice with the medium and incubated for 30 min at 37 °C. PBMCs were co-cultured with labeled K562 cells in specific cell ratios (T/E ratios of 1:1, 1:5, 1:10, 1:20) 96-well plates and incubated under 5% CO_2_ at 37 °C for 12 h in triplicate. The culture supernatants were transferred into a black 96-well plate and fluorescence was measured using a fluorometer (BioTeK, USA) at 490 nm excitation and 520 nm emission wavelengths. Target cells within complete medium and the cells treated with 1% Triton X-100 were considered as negative and positive controls representing spontaneous and maximum fluorescence release, respectively. Cytotoxicity was calculated according to the following formula^34^:

$${\text{Cytotoxicity }}\left( \% \right) = [{\text{test}}\;{\text{release}} - {\text{spontaneous}}\;{\text{release}}]/[{\text{maximum}}\;{\text{release}} - {\text{spontaneous}}\;{\text{release}}] \times 100$$

### Antitumor activity

Fifteen female C57BL/6J mice (6–8 weeks, 20 g) were purchased from the animal resource center (Pasteur Institute of Iran) and maintained under standard housing conditions. The research protocols and all animal studies were approved by the Ethics committee of Pasteur Institute of Iran (IR.PII.REC.1400.034) and followed ARRIVE reporting guidelines^33^. Lymphoblast B lymphocyte TC-1 (#ATCC: CRL-2785) cell line was obtained from the department of Cell Bank (Pasteur Institute of Iran) and cultured in DMEM medium supplemented with 10% Fetal bovine serum (FBS, Sigma) at 37 °C under 5% CO_2_ atmosphere. On day 0, 1 × 10^6^ cells in PBS were injected subcutaneously (*s.c*) into the right flank of the mice and tumor growth was daily examined until the tumor dimension reached 50 mm^3^. After scarification of the mice by cervical dislocation, solid tumor samples were thinly sliced (2 mm thick) and subcutaneously transplanted into the shaved right flank of 15 mice which were anesthetized with intraperitoneal (*i.p*) ketamine (10 mg/ml)/xylazine (10 mg/ml) (Sigma, USA) formulated in water and randomly divided into three groups (5 mice per group). Mutant and wild type groups were injected by the recombinant purified IL-2 proteins diluted in sterile PBS to a final concentration of 1 mg/kg at the tumor site, two times per week for one month. The control group received 200 µl PBS. Tumor size in each group was measured using a caliper and tumor volume was calculated by the following formula: 0.5 × length × width^2^ of the tumor^35^.

### Statistical analysis

Data analysis was performed with GraphPad Prism (v. 8.0). The significance level between two or more groups was determined by two-tailed unpaired t-test and One-way ANOVA analysis of the variance.

## Supplementary Information

Supplementary Information.

## Abbreviations

ConA: Concanavalin A
FDA: Food and Drug Administration
HD-IL-2: High-dose human interleukin-2
IL-2: Human interleukin-2
IL-2R: Interleukin-2 receptor
IFN: Interferon
IPTG: Isopropyl β-d-1-thiogalactoside
Mab: Monoclonal antibody
PBMC: Peripheral blood mononuclear cell
rIL-2: Recombinant wild-type human interleukin-2
SDS-PAGE: Sodium dodecyl sulphate–polyacrylamide gel electrophoresis
TNF: Tumor necrosis factor
Treg: Regulatory T cells
VLS: Vascular leak syndrome
wtIL-2: Wild-type human interleukin-2
