# Supplementary material for: Efficacy and antitumor activity of a mutant type of interleukin 2
Source: Sci Rep. 2022 Mar 30;12:5376. doi: 10.1038/s41598-022-09278-7 (PMC8968711; doi:10.1038/s41598-022-09278-7)

**Efficacy and antitumor activity of a mutant type of interleukin 2**

Rada Dehghan^1#^, Arezoo Beig Parikhani^1#^, Sirous Zeinali^2^, Mohamadali Shokrgozar^3^, Amir Amanzadeh^3^, Soheila Ajdary^4^, Reza Ahangari Cohan^5^, Yeganeh Talebkhan^6*^, Mahdi Behdani^1*^

^1^ Venom and Biotherapeutics Molecules Laboratory, Department of Biotechnology, Biotechnology Research Center, Pasteur Institute of Iran, Tehran, Iran

^2^ Molecular medicine department, Biotechnology Research Center, Pasteur Institute of Iran, Tehran, Iran

^3^ National Cell Bank of Iran, Pasteur Institute of Iran, Tehran, Iran

^4^ Department of Immunology, Pasteur Institute of Iran, Tehran, Iran

^5^ Department of Nanobiotechnology, New Technologies Research Group, Pasteur Institute of Iran, Tehran, Iran

^6^ Department of Biotechnology, Biotechnology Research Center, Pasteur Institute of Iran, Tehran, Iran

**Supplementary Figure S1a.** IPTG Induced bacterial lysate**.** The full size original image of Figure 1a: #1, 2: Lysate of recombinant wild and mutant IL-2 expressing *E. coli* BL21 (DE3) cells before induction; #3, 4: Lysate of *E. coli* BL21 (DE3) expressing wild and mutant IL-2 after induction; M: Protein molecular weight marker.


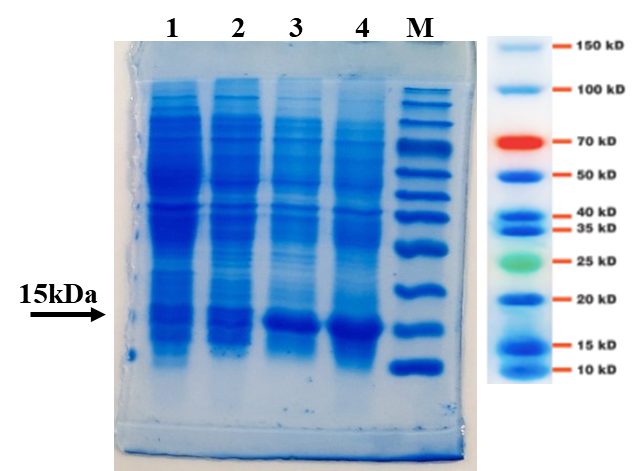


**Supplementary Figure S1b.** Purified Proteins. The full size original image of Figure 1b: #1: Empty well, #2, 3: Purified wild and mutant IL-2 proteins; M: Protein molecular weight marker.


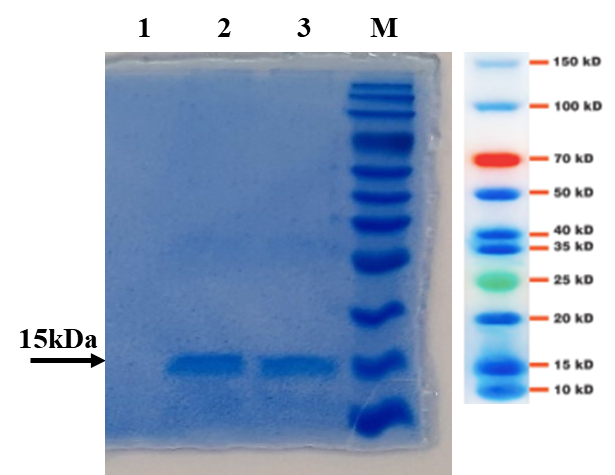


**Supplementary Figure S1c.** **Western blotting analysis.** The full size original image of Figure 1c: #1: His-tagged protein (~30kDa); #2: Lysate of *E. coli* BL21 (DE3) harboring wild IL-2 expression vector before induction (Negative control); #3, 4: Eluted wild and mutant IL-2 proteins; M: Protein Mw marker.


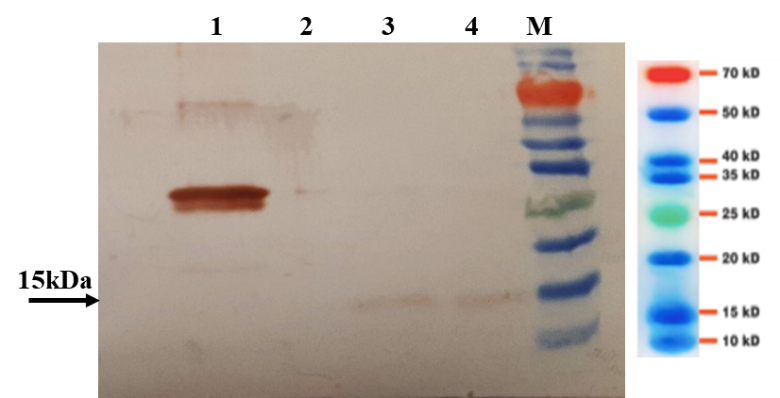

Supplement: Supplementary file 1 — Supplementary Information. [file 41598_2022_9278_MOESM1_ESM.docx]
